# Synthetic Melanin Acts as Efficient Peptide Carrier in Cancer Vaccine Strategy

**DOI:** 10.3390/ijms232314975

**Published:** 2022-11-29

**Authors:** Stefania Cuzzubbo, Benoit Roch, Guillaume Darrasse-Jèze, Benoit Hosten, Manon Leclercq, Nicolas Vignal, Claire Banissi, Eric Tartour, Antoine F. Carpentier

**Affiliations:** 1 Inserm U970, PARCC, Université Paris Cité, 75015 Paris, France; 2 Department of Neurology, Hôpital Saint-Louis, Assistance Publique-Hôpitaux de Paris (AP-HP), 75010 Paris, France; 3 Altevax Inc., Laboratoire de Recherches Biochirurgicales (Fondation Carpentier), Hôpital Européen Georges Pompidou, 75015 Paris, France; 4 Immunology-Immunopathology-Immunotherapy (i3) Laboratory, INSERM UMR-S 959, Sorbonne Université, 75005 Paris, France; 5 Unité Claude Kellershohn, INSERM UMR-S 1144, Hôpital Saint-Louis, Assistance Publique-Hôpitaux de Paris (AP-HP), 75010 Paris, France; 6 Service d’Immunologie Biologique, Hôpital Européen Georges Pompidou, Assistance Publique-Hôpitaux de Paris (AP-HP), 75015 Paris, France

**Keywords:** melanin, L-DOPA, cancer vaccine, adjuvant, nanoparticles, carrier, delivery system, peptide vaccine, dendritic cells, Langerhans cells

## Abstract

We previously reported that a novel peptide vaccine platform, based on synthetic melanin nanoaggregates, triggers strong cytotoxic immune responses and significantly suppresses tumor growth in mice. However, the mechanisms underlying such an efficacy remained poorly described. Herein, we investigated the role of dendritic cells (DCs) in presenting the antigen embedded in the vaccine formulation, as well as the potential stimulatory effect of melanin upon these cells, in vitro by coculture experiments and ELISA/flow cytometry analysis. The vaccine efficiency was evaluated in FLT3-L^−/−^ mice constitutively deficient in DC1, DC2, and pDCs, in Zbtb46^DTR^ chimera mice deficient in DC1 and DC2, and in Langerin^DTR^ mice deficient in dermal DC1 and Langerhans cells. We concluded that DCs, and especially migratory conventional type 1 dendritic cells, seem crucial for mounting the immune response after melanin-based vaccination. We also assessed the protective effect of L-DOPA melanin on peptides from enzymatic digestion, as well as the biodistribution of melanin–peptide nanoaggregates, after subcutaneous injection using [^18^F]MEL050 PET imaging in mice. L-DOPA melanin proved to act as an efficient carrier for peptides by fully protecting them from enzymatic degradation. L-DOPA melanin did not display any direct stimulatory effects on dendritic cells in vitro. Using PET imaging, we detected melanin–peptide nanoaggregates up to three weeks after subcutaneous injections within the secondary lymphoid tissues, which could explain the sustained immune response observed (up to 4 months) with this vaccine technology.

## 1. Introduction

In the last decade, the advent of immune checkpoint inhibitors (ICIs) has led to impressive advances in cancer immunotherapy. Nevertheless, these molecules are efficient only in some cancers, and only in a subset of patients. Therapeutic vaccines represent an attractive strategy for eliciting immune response against cancer cells, and act synergistically with other non-specific immunotherapies, such as ICIs [1,2]. This combinatorial strategy has recently reported encouraging results in early-phase clinical studies [3,4,5]. However, an objective and durable anti-cancer activity is difficult to achieve with the currently available vaccine formulations, especially in the typically immunosuppressed environment of cancer patients [6]. Today, protein/peptide vaccines account for the most widely used approach in cancer vaccine trials [1], but the magnitude of the antigen-specific CD8^+^ T-cell response in patients has been limited so far. The efficacy of subunit vaccines is possibly limited by a scarce delivery of antigens and adjuvants to secondary lymphoid organs, leading to poor immune stimulation or even immune tolerance [7,8]. A common formulation used for peptide vaccines relies on the combination of a water-in-oil emulsion and CpG oligodeoxynucleotide, a Toll-like receptor 9 (TLR9) agonist [9]. However, due to the sustainable antigen release from the depot, water-in-oil emulsions have been reported to sequester specific T cells triggered by the vaccine within the depot itself, thus resulting in exhaustion of T cells and a limited amount of tumor-infiltrated CD8^+^ T lymphocytes [10,11]. Similarly, mRNA vaccines did not induce a sufficient magnitude of CD8^+^ T-cell response, and most often required a step consisting of in vitro stimulation [3].

Nanoparticles represent a new hope in the field of cancer vaccines, since several of them have been proven, in preclinical models, to deliver antigens and adjuvants into lymph nodes (LNs), where cytotoxic immune responses can be mounted by crosstalk between antigen-presenting cells (APCs) and T lymphocytes [1]. We have recently reported successful results with a vaccine platform based on synthetic melanin nanoparticles (L-DOPA melanin), including a complex of peptide–melanin nanoaggregates combined with a TLR9 agonist (CpG oligodeoxynucleotides) [12]. This vaccine formulation proved to be more effective than the combination of incomplete Freund’s adjuvant (IFA) and TLR9 agonists in terms of both the specific T CD8 response and the anti-tumor effect in the E.G7-OVA model [13]. Compared to other nanoparticles, such as carbon nanotubes or metallic nanoparticles, melanin has the advantage of good biocompatibility and biodegradability [14]. However, the mechanisms underlying the adjuvant properties of melanin are still not well known. Some melanins have been reported to possess direct immunomodulatory effects on dendritic cells (DCs), such as polydopamine melanin [15] or natural melanins [16,17].

There are several subsets of professional antigen-presenting cells (APCs) involved in the presentation of antigens from the skin to T cells of the skin-draining lymph nodes. They include Langerhans cells (LCs), which derive from yolk sac and fetal liver macrophage progenitors, and dermal DC1 and dermal DC2, which derive from the common dendritic progenitor CDP in the bone marrow. Dermal DC1 and 2, as well as lymph nodes resident DC1 and DC2 and plasmacytoid DCs (pDCs), are dependent on the ligand on Fms-like tyrosine kinase 3 (FLT3-L) for their differentiation in the bone marrow and their homeostasis in periphery [18]. Consequently, mice constitutively deficient in FLT3-L are devoid of DC1, DC2, and pDCs. If LCs and dermal DCs are important for transporting antigens from the skin to the draining lymph nodes, it has been shown that LN-resident DCs are also essential to prime T cells in the LN, as they can present antigens from LN-migrating skin APCs after phagocytosis or antigen transfer [19], as well as soluble skin antigens migrating to the LNs through the lymphatic vessels.

In this study, we investigated the mechanisms of synthetic L-DOPA melanin as an adjuvant in peptide vaccines. We first analyzed the role of DCs as APCs with this vaccine formulation, and investigated the potential stimulatory effect of melanin upon these cells in vitro. We then evaluated the carrier activity of L-DOPA melanin by assessing its protective effect on peptides, the biodistribution of L-DOPA melanin-peptide nanoaggregates after subcutaneous injection in mice using PET imaging, and the potential role of the different populations of DCs in transportation of nanoaggregates from injection site to lymph nodes and in the activation of T lymphocytes.

## 2. Results

### 2.1. Dendritic Cells Efficiently Phagocytize Melanin–Peptide Nanoaggregates and Cross-Present the Antigen to T Lymphocyte

**Figure 1:**
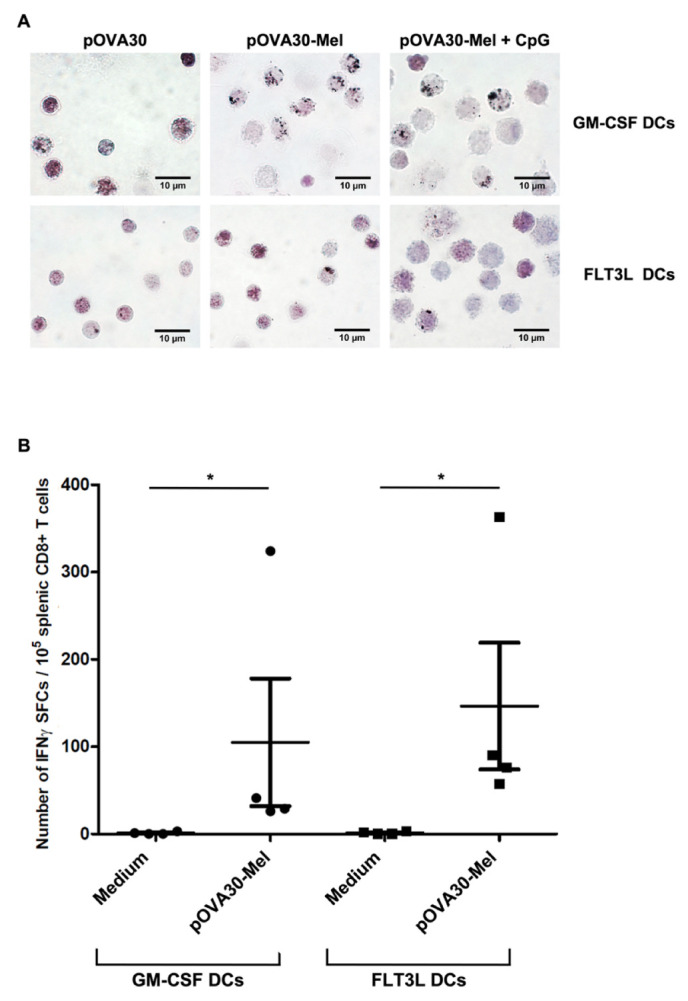
Dendritic cells efficiently phagocytize melanin–peptide nanoaggregates and cross-present the antigen to T lymphocyte. (**A**) Fontana-Masson and hematoxylin–eosin staining of dendritic cells derived from bone marrow cells and cultivated with GM-CSF (GM-CSF DCs) or FLT3L. Low doses of GM-CSF (FLT3L DCs) after 24 h incubation with pOVA30-Mel, pOVA30-Mel, and CpG, or pOVA30 (as negative control). Concentrations used for the different solutions: melanin at 4 μg/mL, pOVA30 at 2 μg/mL, CpG at 1 μg/mL. Experiments were performed twice for each type of cells, and images were taken using an Eclipse 600 Nikon microscope with 100× objective. Intracellular melanin, colored by Fontana-Masson staining, appears as dark dots. The most representative images were chosen for each cell type and condition. (**B**) CD8^+^ splenic T cells isolated from mice vaccinated with [pOVA30-Mel + CpG] (7 days earlier) were co-cultured with bone marrow-derived dendritic cells (GM-CSF or FLT3L DCs) in the presence or absence (medium) of pOVA30-Mel nanoaggregates. Each point represents the mean value of IFNγ SFC in triplicate wells (pooled data from three independent experiments). Bars = mean values ± SEM. * *p* < 0.5.

The role of DCs as APCs has been largely reported in cancer vaccine strategies. Thus, we explored their role in the immune response to melanin-based vaccines. We first investigated whether DCs were capable of phagocyting melanin–peptide nanoaggregates and presenting the antigen to T lymphocytes in vitro. We used bone marrow-derived dendritic cells (BMDCs) by cultivating bone marrow cells with GM-CSF. After 8 days of culture, we incubated BMDCs with melanin–peptide nanoaggregates (or free peptide as negative control) for 24 h, and then stained them with Fontana Masson to observe the intra-cellular melanin. As shown in Figure 1A, BMDCs were capable of internalizing peptide–melanin nanoaggregates. In order to explore all the subpopulations of DCs, we next investigated the melanin internalization by BMDCs cultivated with FLT3L (FLT3L DCs). Indeed, GM-CSF is known to exclusively stimulate proliferation of type 2 conventional dendritic cells (cDC2) [20], whereas FLT3L, in the presence of low doses of GM-CSF, induces the three main subtypes of DCs: type 1 conventional dendritic cells (cDC1, CD11c^+^ CD8a^+^ CD11b^−^), type 2 conventional dendritic cells (cDC2, CD11c^+^ CD8a^−^ CD11b^+^), and plasmacytoid DCs (pDC, CD11c^+^ PDCA-1^+^ CD11b^−^). We used DCs after 7 days of culture to ensure a sufficient amount of the three subtypes [21]. FLT3L DCs were shown to have phagocytic activity on melanin-peptide nanoaggregates, even at a lower rate compared with GM-CSF DCs (average percentage of 2.3% vs. 14.3% of cells, respectively).

Given the synergistic effects observed between CpG and melanin-based vaccine formulation [12], we also explored whether CpG could increase the internalization of melanin by BMDCs. CpG had no significant impact on the potency of BMDCs to exhibit phagocytic activity toward nanoaggregates (Figure 1A).

Next, we wondered whether DCs were also capable of cross-presenting the antigen epitope included in the melanin–peptide complex on their surface. We performed a co-culture experiment, mixing CD8^+^ T cells from mice previously immunized against pOVA epitope (7 days earlier) with BMDCs incubated in the presence (or absence) of melanin–peptide aggregates containing the long peptide (pOVA30-Mel). Both GM-CSF and FLT3L DCs were able to process and cross-present the epitope to T lymphocytes (Figure 1B). As negative control, we performed co-culture wells with T CD8, isolated from mice immunized with [Mel + CpG] without peptide 7 days earlier, and no IFNγ SFCs were observed.

### 2.2. Conventional Dendritic Cells Are Required for Immune Response to Melanin-Based Vaccine

**Figure 2:**
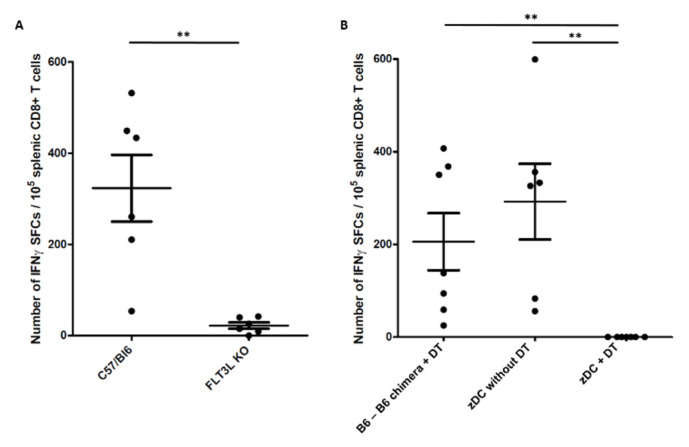
Conventional dendritic cells (cDCs) are required for immune response to melanin-based vaccines. (**A**,**B**) ELISpot analysis of splenocytes from the indicated mouse strain, 7 days after immunization with [gp100-Mel + CpG] vaccine. (**B**) B6-B6 chimera treated with DT and zDC-B6 not exposed to DT represent positive control. Data are presented as the normalized number of IFNγ spot forming cells (SFCs) per 10^5^ CD8^+^ T cells, based on flow cytometry analysis. Each point represents an individual mouse (pooled data from three independent experiments). Bars = mean values ± SEM. ** *p* < 0.01.

We then explored the role of DCs in mounting a specific immune response to melanin-based vaccines in vivo. With this purpose, we used FLT3L^−/−^ mice, lacking all DCs populations (pDCs, cDC1, cDC2). Indeed, dendritic cells require both the receptor tyrosine kinase FLT3 and its ligand FLT3L for their development; however, the loss of FLT3L in mice led to a depletion of DCs larger than the loss of FLT3 [22]. Cytometry analysis of LN cells in FLT3L^−/−^ mice confirmed the depletion of DCs (Figure S1A). We immunized these mice with a melanin-based vaccine against the gp100 antigen, and assessed the specific anti-gp100 CD8 response 7 days later. Mice lacking DCs displayed a dramatic reduction in CD8-specific response compared to C57/Bl6 mice (22 ± 7 vs. 323 ± 73 SFCs/10^5^ CD8^+^ T cells, respectively, *p* < 0.01) (Figure 2A), thus confirming the key role of DCs in triggering CD8^+^ T cells in response to melanin-based vaccines.

We next sought to determine whether cDCs or pDCs were responsible for the immune response to the melanin-based vaccine. For this purpose, we used B6(Cg)-Zbtb46^tm1(HBEGF)Mnz^/J (zDC*^DTR^*) → C57BL/6J chimera mice (zDC mice) which could be cDC-depleted via i.p. injection of DT, while keeping a normal pDC population. Effective depletion in cDCs after DT treatment was checked by cytometry analysis of LN cells (Figure S1B). C57/Bl6 → C57/Bl6 chimera mice (B6-B6 chimera) treated with DT, as well as zDC mice not injected with DT, served as controls. cDC-depletion in zDC mice starting one day before vaccination caused a complete lack of T response after 7 days from the melanin-based vaccine group, compared to the control groups (0 vs. 206 ± 53 (B6-B6 chimera + DT as control) and 292 ± 69 SFCs/10^5^ CD8 T cells (zDC mice without DT as control), *p* < 0.01, Figure 2B). These data thus demonstrate that cDCs have a pivotal role in mounting a specific immune response to melanin-based vaccines.

### 2.3. L-DOPA Melanin Is Not Able to Induce Maturation of Dendritic Cells In Vitro

**Figure 3:**
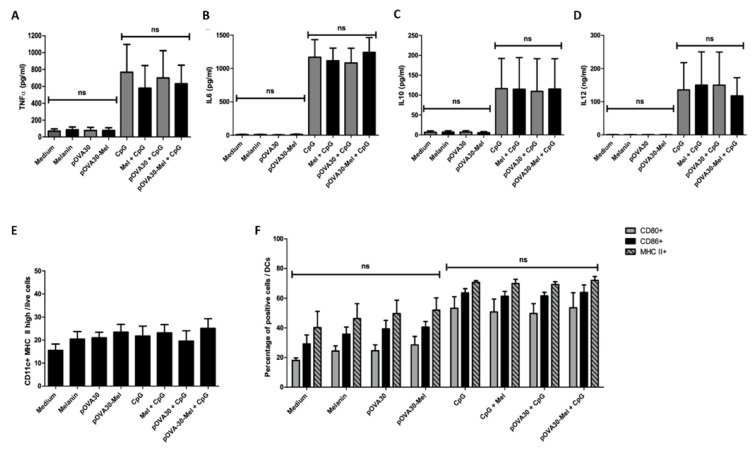
Effects of L-DOPA melanin and pOVA30-Mel nanoaggregates on maturation and activation of GM-CSF DCs. ELISA (**A**–**D**) and flow cytometry analysis (**E**,**F**) of cultures of bone marrow-derived dendritic cells (with GM CSF) after 24 h in vitro incubation with melanin, pOVA30, pOVA30-Mel, CpG; melanin and CpG, pOVA30, and CpG; pOVA30-Mel and CpG; or medium as the negative control (5 × 10^5^ cells/mL; 2 mL/well). Concentrations used for the different solutions: melanin at 4 μg/mL, pOVA30 at 2 μg/mL, CpG at 1 μg/mL. Concentrations of TNFα (**A**), IL-6 (**B**), IL-10 (**C**), and IL-12 (**D**) in supernatants of GM-CSF DCs cultures after 24 h incubation with the different solutions were assessed by ELISA. For gating of cytometry results (**E**,**F**) see Figure S2. Results are presented as mean value ± standard error of the mean (SEM) from three independent experiments; ns = not significant.

**Figure 4:**
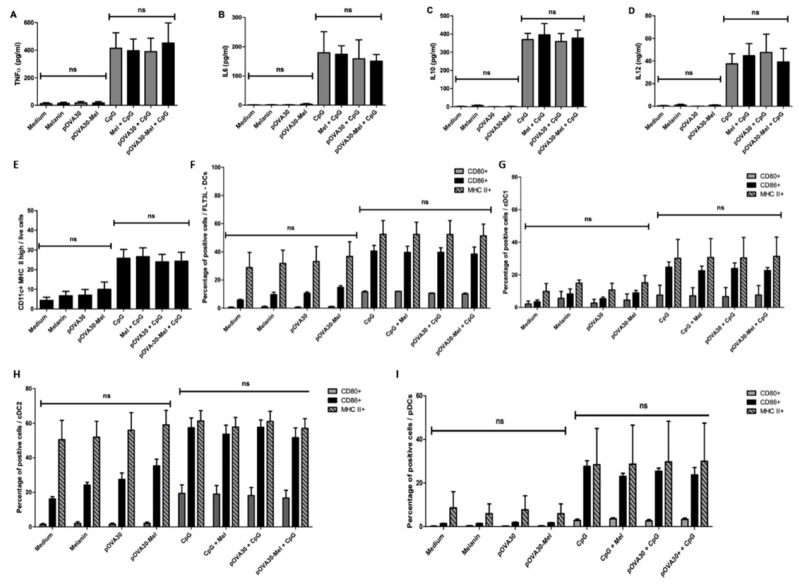
Effects of L-DOPA melanin and pOVA30-Mel nanoaggregates on maturation and activation of FLT3L DCs. ELISA (**A**–**D**) and flow cytometry analysis (**E**,**F**) of cultures of bone marrow-derived dendritic cells (with FLT3L and low dose GM-CSF) after 24 h in vitro incubation with melanin, pOVA30, pOVA30-Mel, and CpG; melanin and CpG, pOVA30, and CpG; pOVA30-Mel and CpG; or medium as the negative control (5 × 10^5^ cells/mL; 2 mL/well). Concentrations used for the different solutions: melanin at 4 μg/mL, pOVA30 at 2 μg/mL, CpG at 1 μg/mL. Concentrations of TNFα (**A**), IL-6 (**B**), IL-10 (**C**), and IL-12 (**D**) in supernatants of FLT3L DCs cultures after 24 h incubation with the different solutions were assessed by ELISA. For gating of cytometry results (**E**–**I**) see Figure S3. Results are presented as mean value ± standard error of the mean (SEM) from three independent experiments; ns = not significant.

The first experiments confirmed to us the crucial role of DCs, and, notably, cDCs, in triggering a specific T response, likely through their APC activity. We then wondered if L-DOPA melanin might also have some immunostimulatory effects on these cells that could potentiate the efficacy of the vaccine, as reported for natural melanin and polydopamine nanoparticles [15,16,17]. With this aim, we performed cytometry and ELISA analysis of BMDCs and their supernatants after 24 h incubation with L-DOPA melanin or pOVA30-Mel nanoaggregates. The activation state was assessed based on the expression of CD80 and CD86 (percentage of positive cells or median fluorescence intensity, MFI) and the release of proinflammatory cytokines (TNFα, IL-6, and IL-12) compared with anti-inflammatory cytokines (IL-10) (Figure 3, Figure 4, Figures S2 and S3). L-DOPA melanin and peptide–melanin nanoaggregates did not exhibit any significant effects on the activation of BMDCs in vitro. We also assessed the percentage of mature cells (CD11c^+^ MHC II^high^ cells/living cells), and no difference was found between the different culture conditions. In FLT3L-cultivated cells, we analyzed specific subpopulations of DCs (DC1, DC2 and pDCs) and a mild, but not statistically significant, increase in the percentage of CD86+ cells in the cDC2 population was found with pOVA30-Mel compared with the negative control (medium): 33.1 ± 2.5% CD86+ cells vs. 15.5 ± 0.8%; *p* = 0.1) (Figure 4 and Figure S3). Notably, there was much less DC1 than DC2 in our culture conditions, and DC1 had a less mature phenotype than DC2 (Figure 4). For both BMDCs (cultivated with GM-CSF or FLT3L), we also explored the effects of melanin and melanin–peptide nanoaggregates in the presence of CpG, and no difference was observed between CpG alone and the combinations of CpG with melanin or melanin–peptide (Figure 3, Figure 4, Figures S2 and S3).

### 2.4. Melanin-Peptide Nanoaggregates Are Carried by Dermal DCs to the Draining Lymph Node

Once the role of DCs in mounting the immune response to melanin-based vaccination was clarified, we investigated the potential carrier effect of L-DOPA melanin. Our first question was how peptide–melanin nanoaggregates reach secondary lymphoid tissues, and specifically, whether they directly enter the lymphatic capillaries or are instead phagocytized and carried by dermal APCs. The latter includes Langerhans cells and dermal migratory dendritic cells, both expressing Langerin (CD207) on their surface. For these experiments, we used transgenic mice (Langerin*^DTR^* mice) which could be depleted of Langerin^+^ cells after DT injection. Effective depletion of Langerin^+^ cells after DT treatment was checked by cytometry analysis (Figure S1C).

**Figure 5:**
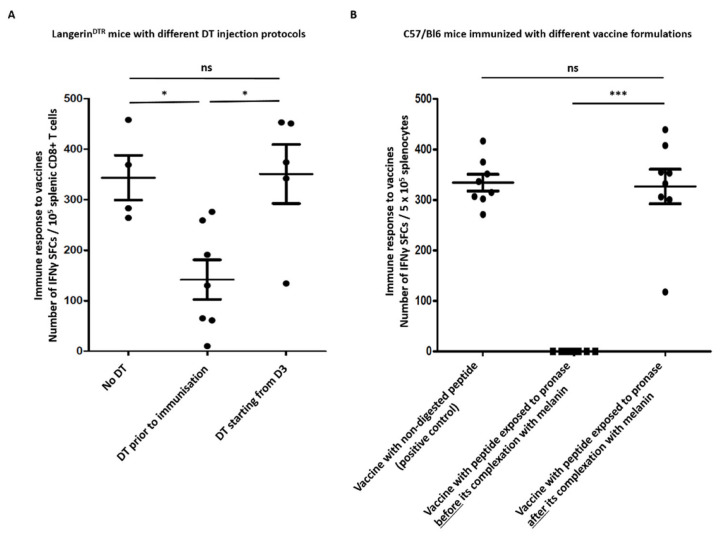
L-DOPA acts as a carrier for peptides, and Langerin^+^ cells carry melanin–peptide aggregates into lymph nodes. (**A**) ELISpot analysis of splenocytes 7 days after immunization with [gp100-Mel + CpG] vaccine in Langerin*^DTR^* mice treated with DT (diphtheria toxin) prior to immunization, 3 days after immunization, or not treated with DT (positive control, mice with Langerin^+^ cells not depleted). Data are presented as normalized number of IFNγ spot-forming cells (SFCs) per 10^5^ CD8^+^ T cells based on flow cytometry analysis. (**B**) ELISpot analysis of splenocytes on day 7, after immunizations on day 0 with different vaccine formulations. Each point represents an individual mouse (n = 8 mice/group with pooled data from 2 different experiments). Bars = mean values ± SEM. * *p* < 0.5, *** *p* < 0.001, ns = not significant.

We immunized these mice with a melanin-based vaccine against gp100, and assessed the specific response in comparison with control mice by ELISpot 7 days later (Figure 5A). When the DT was injected before immunizations and every other day until day 7, the specific T CD8 response was significantly lower in mice lacking Langerin^+^ cells than in control mice (142 ± 32 vs. 344 ± 35 SFCs/10^5^ CD8^+^ T cells, respectively, *p* = 0.01), thus suggesting that a significant part of the melanin–peptide nanoaggregates is transported by Langerin^+^ cells from the injection site into the LN, where the epitope can be presented to T lymphocytes. On the contrary, when the DT was injected 3 days after the immunization, Langerin^+^ cells-depleted mice displayed a similar response compared to control mice (351 ± 40 SFCs/10^5^ CD8^+^ T cells), thus confirming that melanin–peptide nanoaggregates are carried into the LN early, within 3 days after subcutaneous immunization (Figure 5A).

### 2.5. L-DOPA Melanin Protects Peptides from Enzymatic Digestion

**Figure 6:**
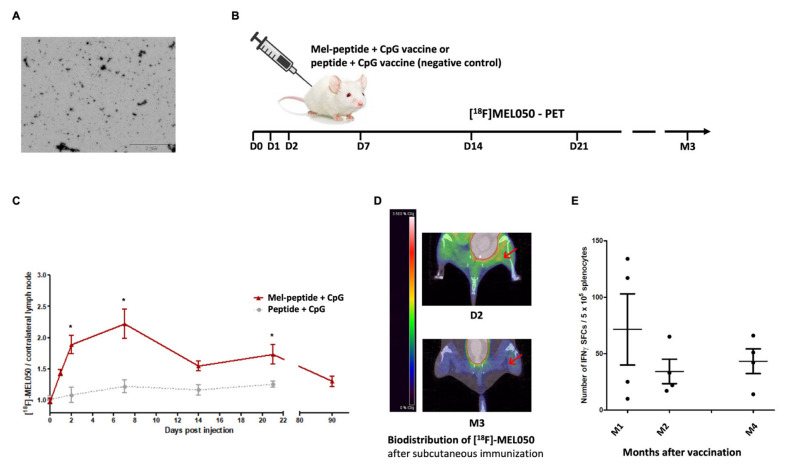
Biodistribution of pOVA30-Mel nanoaggregates after subcutaneous injection in mice. (**A**) Transmission electronic microscopy (TEM) image of peptide–melanin nanoaggregates (gp100). (**B**) Outline of the experiment: Balb/c mice were injected subcutaneously at the right hind leg with [pOVA30-Mel + CpG] or [pOVA30 + CpG] as negative control, and [^18^F]MEL050 CT/PET imaging was performed 1, 2, 7, 14, and 21 days, as well as 3 months later. (**C**) Melanin–peptide kinetics in the right inguinal lymph node post-injection of the vaccine versus negative control, expressed as normalized values of [^18^F]MEL050 (% ID/g) to background radioactivity (contralateral lymph node). (**D**) Representative images of the [^18^F]MEL050_max_ signal into the right inguinal lymph node (red arrow), 2 days and 3 months post-injection of [pOVA30-Mel + CpG. (**E**) ELISpot analysis of splenocytes 1, 2, and 4 months after immunization with [gp100-Mel + CpG] vaccine (injections on days 0 and 14). Results are presented as mean ± standard error of the mean (SEM) (data of four mice per group pooled from two different experiments); Mel = melanin; * = *p* < 0.05.

As previously reported, melanin-based vaccines are prepared by mixing a solution of L-DOPA with a solution of peptide containing the epitope target of the immunization, under aerated and alkaline conditions [12]. In this manner, the peptide binds to the melanin during the L-DOPA polymerization, and peptide–melanin nanoaggregates of an average size (10–20 nm) are obtained (Figure 6A).

To document the role of the NH2 terminal moieties in melanin-binding, we used the VIYRYYGL peptide, either containing or not containing an acetyl residue on the NH2 terminus. We mixed the two peptides with L-DOPA under the same conditions (pH 8.5, under agitation, peptide:L-DOPA ratios from 1:1 to 1:10) and assessed the level of the binding to melanin by SDSpage. Compared to >80% binding of the VIYRYYGL peptide to melanin over a 1:2 ratio, no significant binding (<10%) was seen when the NH2 terminal region was blocked by an acetyl residue (Acetyl-R-VIYRYYGL), pointing out the critical role of the NH2-terminal moieties of peptides in melanin binding (Figure S4A,B).

As efficient binding of the antigenic peptide plays a critical role in obtaining the immunological response to the antigen, we investigated whether L-DOPA melanin protects the peptide from protease degradation, leading to a longer persistence of antigens in vivo after injection. In order to investigate this effect, we incubated pOVA30 peptide as a free peptide or after complexation with L-DOPA melanin (pOVA30-Mel) with pronase, a mixture of endo- and exo-proteases known to cleave both denatured and native proteins, leading to complete digestion into individual amino acids [23,24]. We then used these solutions to immunize mice and, as expected, the free pOVA30 peptide was completely digested (Figure S4C) and mice immunized with it did not develop any T response against pOVA30 (IFNγ ELISpot performed 7 days after injection). On the contrary, mice injected with the vaccine including the peptide exposed to pronase after complexation with L-DOPA melanin developed a strong specific T CD8 response, comparable to those immunized with non-digested vaccine formulation (analysis at day 7, Figure 5B). We thus concluded that L-DOPA melanin fully protects peptides from enzymatic digestion by pronase. To rule out the possibility that L-DOPA melanin could directly inhibit the pronase, we exposed free peptides to pronase in the presence or absence of L-DOPA melanin (not bound to peptides) and performed the SDS page of the sample. In both conditions, the peptides were completely digested. We, therefore, concluded that L-DOPA melanin protects peptides from enzymatic digestion, likely by mechanic inhibition.

### 2.6. Melanin-Based Vaccine Induces a Durable Immune Response

Such a long persistence of the antigen within the lymph node may explain the strength and efficacy of the immune response elicited by the melanin-based vaccine. We then investigated whether this formulation could also induce a durable immune response, which is crucial to achieving significant anti-cancer efficacy. We thus immunized C57/B6 mice (first immunization on day 0, with a boost on day 14) with the melanin-based formulation and assessed the specific T-cell response one, two, and four months later. As shown in Figure 6D, we observed a significant immune response up to four months after immunization.

## 3. Materials and Methods

### 3.1. Peptides and Melanin

The endotoxin-free synthetic pOVA30 (SMLVLLPKKVSGLKQLESIINFEKLTKWTS) and gp100 (KVPRNQDWL) peptides, containing H2-2b epitopes (underlined) [25], and peptides VIYRYYGL and Acetyl-R-VIYRYYGL were purchased from Genosphere Biotechnologies (Paris, France). Vaccine formulations were prepared as previously described [12]. Briefly, pOVA30 or gp100 were mixed with L-DOPA (Sigma-Aldrich, Saint-Quentin-Fallavier, France) in a ratio of 1:2, and the solution was oxidized in aerated conditions. For the experiments exploring the chemical binding between the peptides and the melanin induced by the oxidation process, the different peptides were mixed with L-DOPA in different weight ratios (from 1:1 to 1:10) in the same aerated conditions, and the binding level of the peptides to melanin was assessed by SDS-PAGE.

### 3.2. Animals

Female C57/Bl6JRj and Balb/c mice aged 5 to 7 weeks old were purchased from Janvier Labs (Le Genest-Saint-Isle, France), FLT3L^−/^^−^ mice were breaded in the GDJ facility, and Langerin*^DTR^* transgenic mice were a kind gift from Emmanuel Gautier (Inserm, UMR-S 116, Sorbonne Université, Hôpital de la Pitié-Salpêtrière, Paris, France).

zDC^+/*DTR*^ chimera mice were generated using Zbtb46^tm1(HBEGF)Mnz^ [26] as bone marrow donors, and C57/Bl6 mice as bone marrow chimera recipients. C57/Bl6 mice were irradiated at lethal doses (two doses of 500 cGy, 3 h apart) and intravenously injected with 12 × 10^6^ bone marrow cells 3 h after the second irradiation. 8–10 weeks after lethal irradiation, bone marrow chimeras were reconstituted, and mice were used for experiments. Langerin*^DTR^* transgenic mice were injected i.p. with 40 ng DT (diphtheria toxin, Sigma-Aldrich, Saint Louis, MO, USA) per gram of body weight 24 h before immunizations and then every 48 h. In order to deplete Langerin^+^ cells 3 days after immunization, some Langerin*^DTR^* mice received 40 ng DT per gram of body weight 72 h after immunization. zDC*^DTR^* and C57Bl/6 control chimera mice received 50 ng DT per gram of body weight 24 h before immunization, and then 25 ng DT per gram of body weight every 48 h.

### 3.3. Immunization Protocol

The vaccine was composed of peptide–melanin nanoaggregates (pOVA30-Mel or gp100-Mel) and phosphorothioate oligonucleotide CpG-28 (5′-TAAACGTTATAACGTTATGACGTCAT) (Oligovax, Paris, France), a B-type CpG-ODN [27,28], added just before the immunizations (10 μg/mouse). For some experiments, pOVA30 or pOVA30-Mel nanoaggregates were incubated with proteases (Pronase^®^, Merck Millipore, Burlington, MA, USA), at a ratio of 10:1 at 37 °C and pH 7.5 for 1 h. After incubation, L-DOPA-melanin and CpG-28 or CpG-28 were added to pOVA30 and pOVA30-Mel solutions, respectively, just before the immunizations. All mice received final doses of 25 μg pOVA30, 50 μg L-DOPA melanin and 10 μg CpG-28. Formulations were administered subcutaneously in the right hind leg for PET experiments or in the flank of the mice for the other experiments (100 μL/injection), and mice were sacrificed on the indicated day following the experiments for analysis.

### 3.4. SDS-PAGE

In order to confirm the digestion of pOVA30 peptide by pronase, a Tricine-SDS-PAGE analysis of the different solutions (pOVA30, pOVA30 after digestion, pOVA30 digested in the presence of L-DOPA melanin) was performed as previously described [12].

### 3.5. ELISpot

Splenocytes were prepared as previously described [13] and stimulated at 5 × 10^5^ cells/well at 37 °C in 5% CO_2_ for 21 h with 10 µg/mL of SIINFEKL or KVPRNQDWL antigens (H2-2b epitopes of pOVA and gp100, respectively, with no L-DOPA melanin) using interferon gamma (IFNγ) ELISpot kits (Diaclone, Besançon, France). Cells were also stimulated with 100 ng/mL phorbolmyristate acetate and 1 ng/mL ionomycin (Sigma-Aldrich) as a positive control, and then cultured with an irrelevant epitope as a negative control. IFNγ spot-forming cells (SFCs) were counted on a Cellular Technology Ltd. (Shaker Heights, OH, USA) ELISpot reader using 5.0.3 software. The results are presented as the difference of the average spot count of triplicate experimental wells and the average spot count of duplicate negative control wells. For co-culture experiments, CD8^+^ splenic T cells, isolated from pOVA30 immunized mice (or not as control), were cultured (10^5^ cells/well) in duplicate with dendritic cells (4 × 10^5^ cells/well), either in the presence or not of pOVA30-Mel nanoaggregates (final concentrations of melanin and peptide at 4 and 2 µg/mL, respectively).

### 3.6. Bone Marrow Isolation and Generation of Dendritic Cells

Femurs and tibiae from C57/Bl6 mice were removed and debrided from muscles. Intact bones were disinfected in 70% ethanol and washed, and then both ends were cut to flush out the marrow. Dendritic cells (DCs) were generated as previously described, with some modifications [21]. Briefly, bone marrow cells were cultured in 100 mm bacteriological petri dishes with high glucose RPMI-1640 or Iscove’s modified Dulbecco’s medium, supplemented with 10% heat-inactivated fetal bovine serum (Eurobio, Les Ulis, France), 2 mM l-glutamine, 0.05 mM 2-mercaptoethanol, penicillin (100 U/mL), and streptomycin (100 μg/mL). Cells were seeded at 2 × 10^6^ cells in a medium containing 20 ng/mL murine GM-CSF (granulocyte-macrophage colony-stimulating factor) for the generation of GM-CSF DCs, or at 1 × 10^7^ cells in medium containing 200 ng/mL human FLT3L (fms tyrosine kinase 3 ligand) and 300 pg/mL murine GM-CSF for the first 6 days and 1 ng/mL for FLT3L DCs. GM-CSF DCs and FLT3L DCs were used for experiments after 7 or 8 days of culture, respectively [29]. Only non-adherent cells were used for experiments. All cytokines were obtained from PeproTech (Cranbury, NJ, USA).

### 3.7. Incubation of Dendritic Cells with Melanin and Fontana-Masson Staining

Both GM-CSF DCs and FLT3L DCs were incubated for 24 h at 37 °C. This was conducted at 5% CO_2_ in 12-wells plates at 5 × 10^5^ cells/well, in a volume of 2 mL/well with the indicated solutions. Solutions were used at the following concentrations: 4 μg/mL melanin nanoparticles, 2 μg/mL pOVA30, 1 μg/mL CpG. After incubation, cells were washed, transferred into Superfrost Plus slides (VWR^®^ Superfrost^®^ Plus Micro Slides), and stained with Fontana-Masson by using a stain kit (Sigma Aldrich, Saint Louis, MO, USA) following the manufacturer’s recommendations. Hematoxylin–eosin staining was then performed, and slides were washed and covered with cover slip (Marienfield, Germany) by using a mounting medium (Dako, Santa Clara, CA, USA). Images were obtained using a Nikon’s Eclipse E600 upright microscope with 100× objective. The degree of melanin phagocytosis by cells is presented as the mean percentage of cells internalizing melanin or pOVA30-Mel nanoaggregates in two independent experiments, where the percentage is calculated as the number of cells with melanin in 10 random images (100×) divided by the whole number of cells, for each condition.

### 3.8. ELISA Analysis

Concentrations of Tumor Necrosis Factor α (TNFα), Interleukin-6 (IL-6), Interleukin-10 (IL-10) and Interleukin-12 (IL-12) were determined by ELISA (PeproTech kits) in supernatants of cell cultures (bone marrow-derived GM-CSF DCs and FLT3L DCs) after 24 h incubation with the different solutions, following the manufacturer’s recommendations. The absorbance was measured at 450 nm. The level of cytokines in culture supernatants was calculated relative to the standard sample of the different dilutions and expressed as concentration in supernatant cultures. The results are presented as the average concentration, calculated in triplicate wells, for each condition.

### 3.9. Flow Cytometry Analysis

Cells were incubated with anti-mouse CD16/CD32 (clone 93, Biolegend, San Diego, CA, USA) at 4 °C for 15 min, and then stained (1 × 10^6^ cells/well at 4 °C for 30 min) with different combinations of Amcyan LIVE/DEAD Fixable Aqua Dead Cell Stain Kit (Invitrogen, Waltham, MA, USA) and the following Biolegend anti-mouse conjugated antibodies: APC anti-CD11c (clone N418), APC anti-MHC II (clone M5/114.15.2), Alexa Fluor 700 anti-CD11b (clone M1/70), Pacific Blue anti-CD8α (clone 53-6.7), FITC anti-CD8α (clone 53-6.7), BV650 anti-CD86 (clone GL-1), PE-Cyanine 7 anti-CD86 (clone GL-1), Pacific Blue anti-PDCA-1 (clone 129C1), BV785 anti-Ly-6c (clone HK1.4), PECy7 anti-CD207 (clone 4C7), Pacific Blue anti-CD3 (clone 17A2), BV711 anti-CD4 (clone GK1.5); PerCP Cy5.5 anti-CD19 (clone 3D6112), eBioscence: PE anti-MHC II (clone NIMR-4), FITC anti-CD80 (clone 16-10A1), BD Pharmigen: BV711 anti-CD103 (clone M290), PE anti-CD11b (clone M1/70), PerCP Cy5.5 anti-CD3 (clone 17A2), PerCP Cy5.5 anti-NKP46 (clone 29A1.4), or Invitrogen: APCe780 anti-CD8α (clone 53-6.7). For some experiments, we used biotin anti-CD8α (clone 53-6.7, Biolegend) at 4 °C for 30 min, and then BV711 streptavidin (Biolegend). Isotype controls were included in each experiment. Acquisitions were performed on BD LSRII and data were analyzed with FlowJo software (Treestar).

### 3.10. [^18^F]MEL050 PET/CT Imaging

As previously described [30], automated radiosynthesis of the radiotracer [^18^F]MEL050 was performed on a trasis AllinOne^TM^ synthesis module. The radiotracer was then injected via the tail vein (10.8 ± 1.1 MBq in a volume of 150 μL) at the indicated day after vaccination in Balb/c mice (pOVA30-Mel + CpG). Mice were placed in the PET camera in prone position, and a CT acquisition was performed just before PET acquisition in order to anatomically localize PE signals. PET images were then acquired (60 min after the injection of the tracer) by using an Inveon microPET/CT scanner (Siemens Medical Solutions), designed for small laboratory animals. Both [^18^F]MEL050 and PET/CT imaging were performed under anesthesia (isoflurane/oxygen, 2.5% for induction at 0.8–1.5 L/min and 1.5% at 0.4–0.8 L/min thereafter). The spatial resolution of the Inveon PET device was 1.4 mm full-width at half-maximum (FWHM) at the center of the field of view (FOV). Images were reconstructed using a two-dimensional ordered subset expectation maximization (Fourier rebinning/2-D OSEM) method, including corrections for dead scanner time, scatter radiations and randoms. Quantification analysis of PET/CT images was performed by drawing volumes of interest in organs for biodistribution studies. All values of radioactivity concentrations were normalized by the injected dose, and expressed as percentage of the injected dose per g of tissue (% ID/g). Results are presented as ratios with contralateral radioactivity (background signal).

### 3.11. Statistics

Statistical analyses were performed using Prism 5.0 (Graphpad software^®^). Based on the type of variable (continuous, normally distributed, non-parametric), data were analyzed with the Mann–Whitney test, paired Student’s test, or one-way ANOVA (with Bonferroni correction). All reported *p* values are based on two-sided tests with a minimum significance level of 0.05: * *p* < 0.05, ** *p* < 0.01, *** *p* < 0.001.

## 4. Discussion

Nanoparticle carriers are an attractive approach for cancer vaccines because they can easily reach the secondary lymphoid organs, specifically APCs, and trigger CD8+ T-cell response. Several nanovaccines showed promising results in preclinical studies, such as metallic nanoparticles (gold or γFe_2_O_3_), carbon nanotubes, and cationic polymers [1]. However, the move from bench to bedside is still hampered by toxicity issues for most of these nanoparticles. Compared to these, melanin has the important advantage of being biocompatible and biomimetic. In addition, synthetic melanin possesses a high loading efficiency and a possibility of manipulating both the binding and release of molecules [31]. All of these features make melanin an ideal candidate for drug delivery systems, especially in cancer vaccine strategies. Our peptide vaccine, based on L-DOPA melanin, had been proven to trigger potent cytotoxic responses and significantly suppress tumor growth in mice [12,13], but the mechanisms of L-DOPA melanin as an adjuvant in vaccine remained unclear.

In this study, we first explored the role of dendritic cells in mounting the immune response after vaccination with melanin-based formulation in mice. In vitro studies showed that DCs are capable of processing melanin–peptide nanoaggregates and presenting the antigen to T lymphocytes. Immunization studies in mice lacking DCs (FLT3L^−/−^) confirmed that these cells are crucial to mounting the immune response. Experiments using cDC-depleted mice (DT-treated zDC-DTR chimera mice) particularly demonstrated the essential role of conventional DCs in triggering a T-specific response to melanin-based vaccine. Our results are in line with prior reports on protein/subunit vaccines. A pivotal role was especially reported for skin-derived migratory DCs in antigen presentation in several vaccine formulations (including Alum, AS01, or CpG as adjuvants) [31,32,33,34]. Unfortunately, the intrinsic photosensitive properties of melanin limit the use of fluorescent dyes to follow the phagocytosis of melanin–peptide complexes, and, thus, to define precisely which cell type presents the antigen to T lymphocytes within the lymph node. Cytometry analysis of the main APC populations (macrophages, migratory and resident cDC1, cDC2, and pDCs) within the draining lymph nodes 2 or 14 days after vaccination did not show any significant difference in recruitment or activation between mice vaccinated with the melanin-based formulation and the controls (peptide + CpG) (data not shown). However, the depletion of dermal APCs (Langerin^+^ cells) significantly impacted the specific response to the melanin-based vaccine, even if less drastically than complete ablation of cDC1 or cDC2 in the FLT3L^−/−^ and zDC*^DTR^* models. These results in different transgenic mouse strains suggest that FLT3^+^ CD103^+^ CD207 (Langerin)^+^ dermal migratory cells 1 (mDC1)—which are absent in all three FLT3L^−/−^, zDC*^DTR^*, and Langerin*^DTR^* models—express phagocytic activity on the melanin–peptide nanoaggregates at the site of injection, carry them within the draining LN, and then present the antigen to T cells. However, we also showed that cDC2 (GMCSF DCs) are capable of phagocyting the melanin–peptide nanoaggregates and presenting the antigen to T lymphocytes in vitro (Figure 1), thus prospecting another possible scenario where resident cDC2 in lymph nodes participates in triggering CD8^+^ T cells.

Given the immunomodulatory effects reported for other melanins, we explored whether L-DOPA melanin had similar properties upon BMDCs. Unlike other types of melanin [15,16,17,35], L-DOPA did not exhibit any effect on maturation or activation of BMDCs, despite it exhibiting active phagocytosis of melanin. Under the hypothesis that L-DOPA melanin might stimulate dendritic cells other than cDC2, we also investigated melanin’s effects on FLT3L BMDCs in order to include all three subpopulations of DCs (cDC1, cDC2, and pDCs), but L-DOPA melanin and peptide–melanin nanoaggregates did not demonstrate any significant effect on maturation or activation in any of the three types of DCs. With regard to cDC1, this subset of cells was very small in conditions without CpG compared with those with CpG (0.8–1.5% vs. 24.5–27.4%, respectively). Our results are in line with Kirkling et al., who reported that cDC1 cells developed by FLT3L culture are not mature in the absence of Noch3 pathway activation [20]. However, 24 h incubation with melanin nanoparticles or pOVA30-Mel nanoaggregates was not able to stimulate maturation in these cells, and no additive effect by melanin was seen in conditions with CpG. This different effect of synthetic L-DOPA melanin compared with other melanins may be easily explained in the case of natural melanins, as the latter are commonly non-purified. In addition, molecules other than melanin are present and could have some immunostimulatory properties [16]. As for polydopamine nanoparticles, they mainly differ from L-DOPA melanin in size (200–250 nm vs. 10–20 nm), and phagocyting bigger particles might have diverse effects on cells. On the other hand, the greater size of polydopamine particles might represent a disadvantage in terms of ease of lymphatic draining towards LN.

The carrier properties of L-DOPA melanin for peptides were shown by in vitro and in vivo experiments. L-DOPA melanin fully protected peptides from enzymatic digestion, a known cause of limited effectiveness of peptide/subunit vaccines [1]. In addition, [^18^F]MEL050 PET showed that peptide–melanin nanoaggregates quickly reached the inguinal LN, and were still detected up 21 days post-injection in mice. Such a long persistence within the secondary lymphoid tissues could explain the prolonged and sustained immune response to the vaccine (up to 4 months after vaccination). PET imaging also showed that L-DOPA melanin did not diffuse into organs or tissues other than the injection site and the draining LN. This biodistribution contrasts with other nanoparticles, such as gold nanoparticles and carbon nanotubes, which might accumulate in several organs and induce toxicity. However, a limitation of the PET study is that [^18^F]MEL050 binds melanin, and not the bound peptide. We can, therefore, only assume that peptide remains bound to melanin within the LN. Our data suggest that L-DOPA melanin is more efficient than polydopamine in carrying peptides to LNs and prolonging their retention, since Wang et al. reported polydopamine–peptide retention into draining LNs only up to 48 h after injection [15]. We hypothesized that this difference could be related to the different sizes of L-DOPA melanin–peptide (10–20 nm diameter) and polydopamine–peptide nanoaggregates (230 nm), since nanoparticles ranging from 20 to 200 nm more easily enter the lymphatic capillaries, and remain longer in the LNs than larger nanoparticles [36]. Other authors reported that very small particles (<25 nm in diameter) are those that best reach the lymph nodes [37], and this size corresponds to L-DOPA melanin nanoparticles [12]. With this hypothesis, we assessed the specific CD8 response to the melanin-based vaccine in mice lacking dermal migratory APCs (Langerin^+^ cells): dermal DCs (migratory cDC1) and Langerhans cells. Interestingly, the specific T CD8 response was significantly lower in mice lacking Langerin^+^ cells, thus suggesting that a significant part of the melanin–peptide nanoaggregates is transported by dermal cells from the injection site to the LN. Furthermore, the depletion of migratory APCs three days after the immunization did not affect the specific immune response, thus confirming that peptide–melanin nanoaggregates are carried early within the LN (Figure 5A).

## 5. Conclusions

Herein, we demonstrated that L-DOPA melanin acts as an efficient carrier for peptides by fully protecting them from enzymatic digestion, a known cause of the limited effectiveness of prior peptide vaccines. Migratory dermal cDC1 dendritic cells likely play a pivotal role in mounting the immune response to the melanin-based vaccine. Results from transgenic mice experiments indeed suggest that these cells carry the melanin–peptide nanocomplexes to the draining lymph node, and that they process and cross-present the antigen to T lymphocytes, either directly or via a LN-resident APC.

## 6. Patents

The AP/HP (Assistance Publique de Hopitaux de Paris) holds a patent position on this vaccine technology. A.F. Carpentier and C. Banissi are listed as inventors.

## Data Availability

All data concerning this work are included in this report and Supplementary Data File.

## Supplementary Materials

The following supporting information can be downloaded at: https://www.mdpi.com/article/10.3390/ijms232314975/s1, Figure S1: Lymph node depletion of dendritic cells in FLT3L^−/−^ mice and zDC^DTR^ chimera after DT injection and Langerin+ cells in Langerin^DTR^ mice; Figure S2: Effects of L-DOPA melanin and pOVA30-Mel nanoaggregates on maturation and activation of GM CSF dendritic cells; Figure S3: Effects of L-DOPA melanin and pOVA30-Mel nanoaggregates on maturation and activation of FLT3L DCs; Figure S4: L-DOPA melanin protects pOVA30 peptide from enzymatic digestion.
