# Supplementary material for: Synthetic Melanin Acts as Efficient Peptide Carrier in Cancer Vaccine Strategy
Source: Int J Mol Sci. 2022 Nov 29;23(23):14975. doi: 10.3390/ijms232314975 (PMC9738778; doi:10.3390/ijms232314975)
Supplement: Supplementary file 1 [file ijms-23-14975-s001.zip › ijms-2014297-supplementary.pdf]

# Supplementary figures

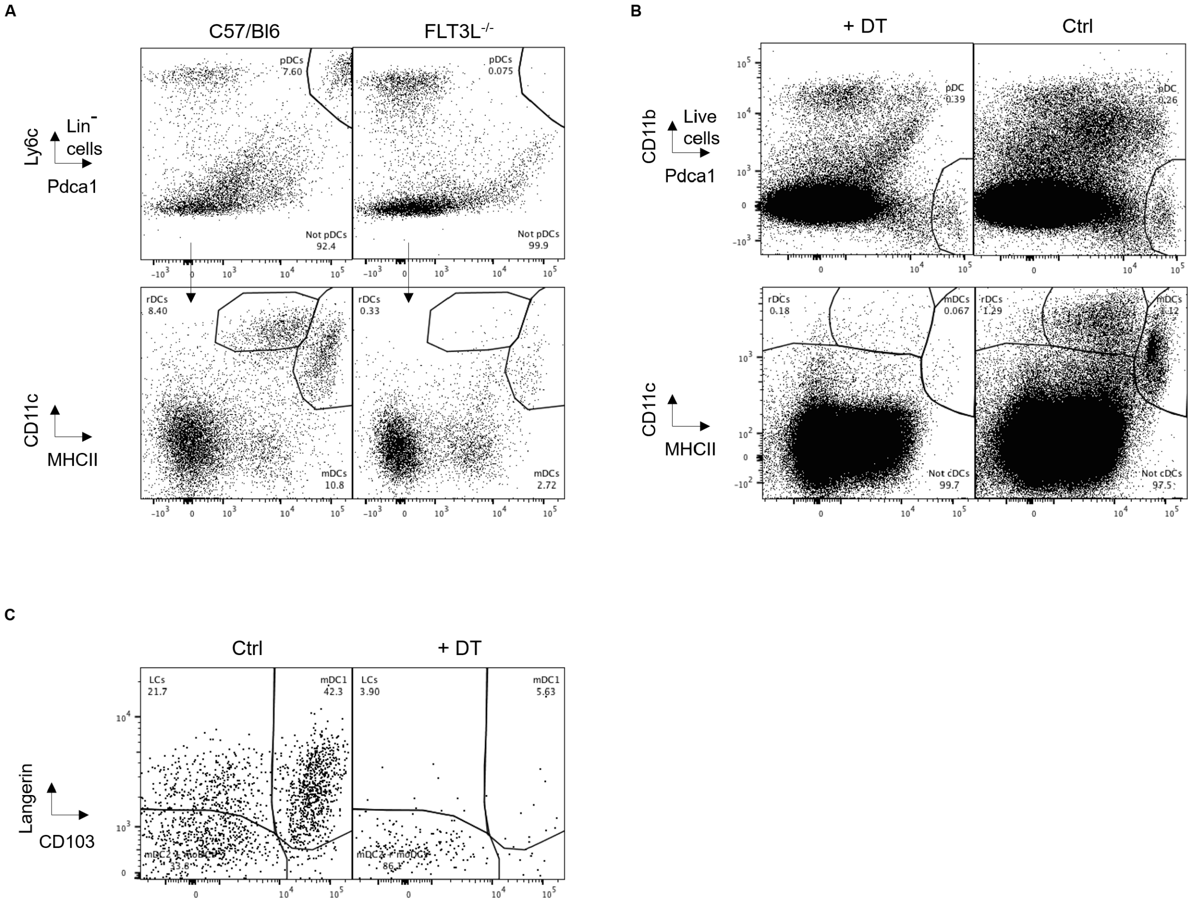


**Figure S1. Lymph node depletion of dendritic cells in FLT3L$^{-/-}$ mice and zDC$^{DTR}$ chimera after DT injection and Langerin$^{+}$ cells in Langerin$^{DTR}$ mice.**
Cytometry analysis of lymph node cells in FLT3L-/- mice and control mice (A) and zDC$^{DTR}$ chimera 24 hours after 1 injection of DT (B). (C) Depletion of Langerin+ cells in Langerin$^{DTR}$ mice after DT injection compared to control mice without DT injection.

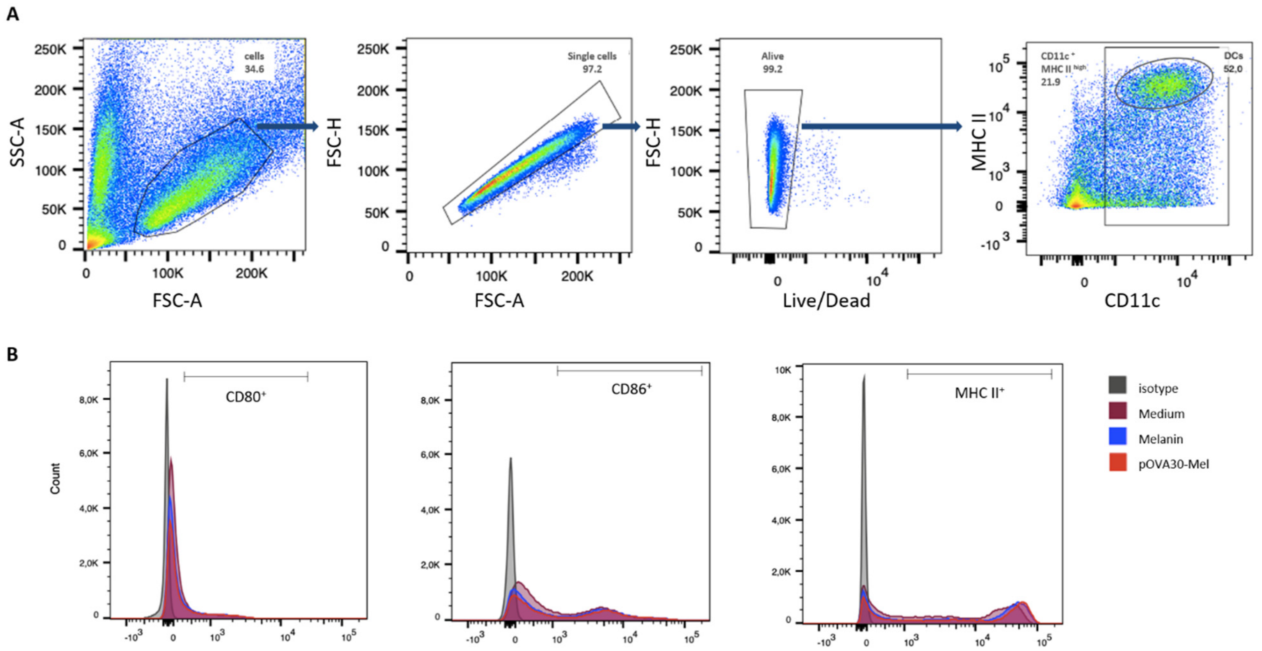


**Figure S2. Effects of L-DOPA melanin and pOVA30-Mel nanoaggregates on maturation and activation of GM CSF dendritic cells.**
Cytometry analysis of bone marrow derived dendritic cells (by GM CSF) after 24 hours incubation with melanin, pOVA30-Mel, or medium as negative control (5 x $10^5$ cells/mL; 2 mL/well). Concentrations used for the different solutions: melanin at 4 µg/mL, pOVA30 at 2 µg/mL, CpG at 1 µg/mL. (A) Cells were gated on living cells or living CD11c$^+$ cells to assess the percentage of mature dendritic cells. (B) Representative MFI of CD80+ CD86+ or MHC II+ cells in the indicated conditions.

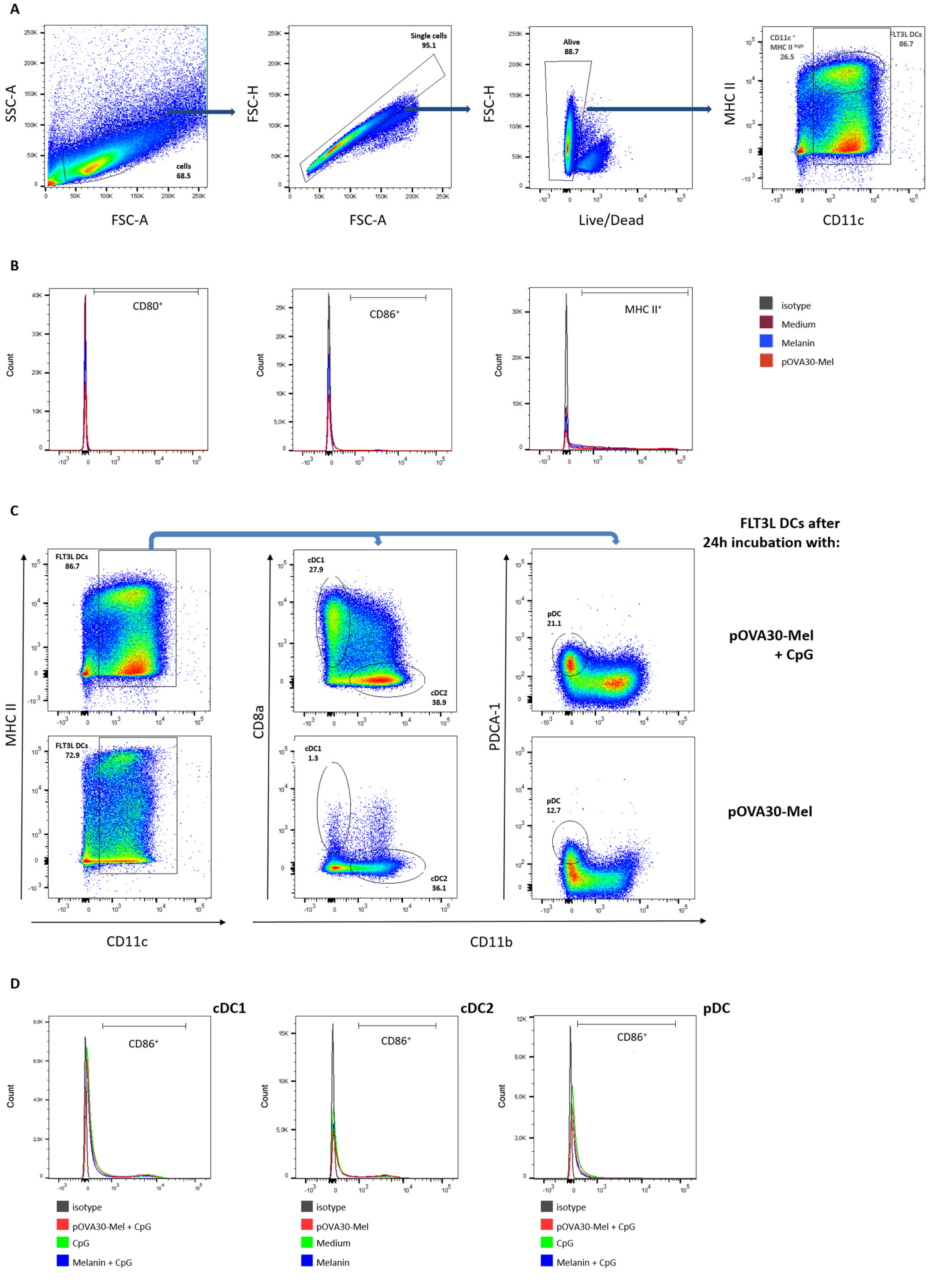


**Figure S3. Effects of L-DOPA melanin and pOVA30-Mel nanoaggregates on maturation and activation of FLT3L DCs.**

Cytometry analysis of cDC1, cDC2 and pDCs (FLT3L DCs) after 24 hours incubation with melanin, pOVA30, pOVA30-Mel, CpG, melanin and CpG, pOVA30 and CpG, pOVA30-Mel and CpG or medium as negative control (5 x $10^5$ cells/mL; 2 mL/well). Concentrations used for the different solutions: melanin at 4 µg/mL, pOVA30 at 2 µg/mL, CpG at 1 µg/mL. (A) Cells were gated on living cells or living $CD11c^+$ cells to assess the percentage of mature dendritic cells or the expression of CD80, CD86 and MHC II as MFI (B). (C) Cells were gated on living double positive $CD11c^+$ $CD8\alpha^+$ and $CD11b^-$ cells (cDC1), double positive $CD11c^+$ $CD11b^+$ and $CD8\alpha^-$ cells (cDC2) or double positive $CD11c^+$ PDCA-$1^+$ and $CD11b^-$ cells (pDC) to assess the expression of CD80, CD86 and MHC II for each subpopulation (D). (B) and (D) report representative MFI of CD80+ CD86+ or MHC II+ cells in the indicated conditions and cell subtype.

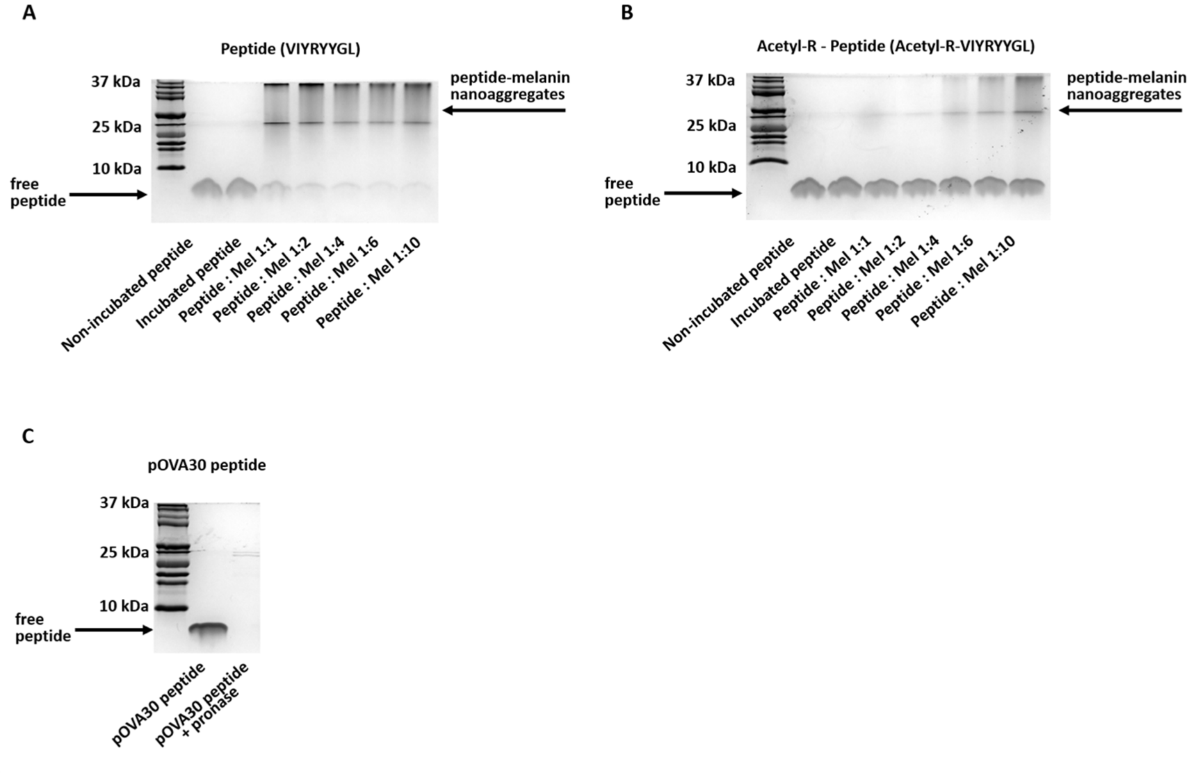


**Figure S4.** L-DOPA melanin protects pOVA30 peptide from enzymatic digestion

SDS-PAGE showing the migration of the indicated peptide within the resolving gel. (A, B) SDS-PAGE showing the peptide remaining (not bound to melanin, indicated as free peptide) after polymerization of L-DOPA at the indicated ratio peptide:melanin. The peptide-melanin nano-aggregates remain at the first phase of gel. Not-incubated peptide (extemporary) and incubated peptide (at the same conditions of the other solutions but without L-DOPA solution) represent controls. (C) SDS-PAGE showing the migration of pOVA30 peptide and its full digestion after incubation with pronase (peptide not visible).
